# Global Distribution and Natural Recombination of Hepatitis D Virus: Implication of Kyrgyzstan Emerging HDVs in the Clinical Outcomes

**DOI:** 10.3390/v14071467

**Published:** 2022-07-02

**Authors:** Amina Nawal Bahoussi, Pei-Hua Wang, Yan-Yan Guo, Nighat Rabbani, Changxin Wu, Li Xing

**Affiliations:** 1 Institutes of Biomedical Sciences, Shanxi University, 92 Wucheng Road, Taiyuan 030006, China; minabau28@gmail.com (A.N.B.); wangph201@gmail.com (P.-H.W.); guoyy101@gmail.com (Y.-Y.G.); fanguo0096@gmail.com (N.R.); cxwu20@sxu.edu.cn (C.W.); 2 The Key Laboratory of Medical Molecular Cell Biology of Shanxi Province, Shanxi University, 92 Wucheng Road, Taiyuan 030006, China; 3 Shanxi Provincial Key Laboratory for Prevention and Treatment of Major Infectious Diseases, 92 Wucheng Road, Taiyuan 030006, China

**Keywords:** *Deltavirus* genus, HDV RNA, viroid, genotype, phylogenetic analysis, genomic recombination

## Abstract

Discrepancies in human hepatitis delta virus (HDV) genotypes impact the virus’ biological behavior, clinical manifestation, and treatment response. Herein, this report aims to explore the role of recombination in the worldwide genotypic distribution and genetic diversity of HDV. Three-hundred-forty-eight human HDV full-length genomic sequences of ~1678 nt in length, isolated in twenty-eight countries worldwide between 1986 and 2018, were analysed. Similarity analysis and recombination mapping were performed, and forty-eight recombination events were identified, twenty-nine of which were isolated from Kyrgyzstan and determined to be involved in the diversity and extension of HDV sub-genotypes. HDV recombination occurred only between the genetically close genotypes (genotype 5 and genotype 2) or mainly within genotype 1, suggesting the complex replicative molecular mechanisms of HDV-RNA. The global distribution and classification of HDV genotypes have been updated, indicating that HDV recombination is one of the driving forces behind the biodiversity and the evolution of human HDV genomes. The outcome analysis suggests that the expansion of HDV sub-genotypes and the complex recombination networks might be related to the genomic character of Kyrgyzstan circulating strains and extensive mobility within countries and across borders. These findings will be of great importance in formulating more effective public health HDV surveillance strategies and guiding future molecular and epidemiological research to achieve better clinical outcomes.

## 1. Background

Infectious hepatitis, including hepatitis A, B, C, D, and E, are caused by five distinct hepatotropic viruses, which constitute a major global health concern by their genetic diversity and transmission routes [1]. Hepatitis D infection (HepD), the most severe form of human viral hepatitis, is caused by the hepatitis delta virus (HDV) and requires the assistance of a circulating hepatitis B virus (HBV) to complete its replication [2]. The coinfection of HBV and HDV may engender liver failure, accelerate liver fibrosis, and increase the decompensation of liver cirrhosis compared to HBV mono-infection [3]. Approximately 5% of the worldwide chronic HBV carriers were estimated HDV infected [4], or one in five cases of hepatocellular carcinoma and liver disease are individuals diagnosed as HDV/HBV co-infected [4,5], indicating the substantial contribution of HDV in liver injury.

Hepatitis delta virus is the smallest and most unique satellite human virus [2]. HDV is the only reported member of a unique *Deltavirus* genus in the single *Kolmioviridae* family within the new realm *Ribozyviria* [6]. HDV was first identified by Rizzetto et al. in 1977 in Turin, Italy, in sera and liver biopsies of patients with advanced hepatitis B [7]. HDV infection occurs only in HBV-infected individuals where HDV uses the HBV-encoded surface antigens (HBsAg) to complete its life cycle and fulfil virus transmission [2].

HDV infection is endemic in Western and Central Africa, Mongolia, and the Republic of Moldova [4]. HDV infection has also been observed in many countries of Europe [8,9], the Eastern Mediterranean Region [10], the Brazilian Amazon [11], and Sub-Saharan Africa [12]. Turkey [13], Iran [14], and the Pacific Islands of Kiribati [15] also revealed a high HDV prevalence.

HDV virion is ~36 nm in diameter, composed of an envelope (containing the HBsAg) and a Delta ribonucleoprotein (RNP) complex. HDV RNP contains a negative-sense circular RNA genome of ~1.7 kb in length, bearing a single open reading frame (ORF) that encodes only one protein called HDV antigen (HDAg) [16,17]. Although HDV is conditioned by HBsAg, the synthesis of its RNA genome is independent of HBV, using the host cellular enzymes [18]. The HDV genome is somewhat similar to plant viroids [19] and replicates through a rolling circle process by the RNA-directed RNA synthesis without DNA intermediate [20]. The HDV mRNA transcription and genomic RNA synthesis are mediated by the host RNA polymerase II, while the antigenomic RNA is reported to be mediated by RNA polymerase I, which remains controversial [21,22]. The genomic and antigenomic RNAs hold a self-cleaving activity mediated by an RNA element called “ribozyme” that can cleave the genomic RNA at the nt 685/686 position or antigenomic RNA at the nt 900/901 position [23]. As a defective virus, HDV requires the S, M, and L-HBsAg proteins of HBV to generate its envelope, hence propagating [24]. The HDV-encoded HDAg protein occurs in two forms, the small HDAg (S-HDAg, 24 kDa) and the large HDAg (L-HDAg, 27 kDa). Both protein forms derive from the same HDV mRNA [25,26] by a single nucleotide conversion in the UAG stop codon for S-HDAg to UGG (A–G), leading to L-HDAg [27,28]. The S-HDAg is crucial for viral replication and RNA translocation [29], whereas L-HDAg is involved in the viral particle assembly [30].

HDV was initially classified into three genotypes and then extended to eight major genotypes [31]. The eight HDV genotypes have been updated and defined using phylogenetic analysis (HDV-1 to HDV-8) with different clinical outcomes and specific geographical distribution of particular genotypes [32,33,34]. Le Gal et al. updated the HDV phylogenetic genotyping based on partial genomic regions from patients newly diagnosed with HDV between 2001 and 2014 [32]. Since 2014, more strains have been isolated and reported, among which HDV-2, which has emerged recently in Egypt, and HDV-3 in the Amazon region of South America [35].

Clinically, HDV-1 is reported to engender mild to severe stages of liver disease. For instance, a study conducted on the population of Taiwan reported more serious liver damage in HDV-1 patients compared to HDV-2, with outcomes ranging from cirrhosis and HCC to death [36]. HDV-2 is reported to cause mild-stage disease with less damage than HDV-1 [37]. HDV-4 patients exhibited clinical outcomes relatively similar to HDV-2 (HDV-2b), while HDV-3 infection is correlated with more adverse outcomes and fulminant forms [38]. The clinical behavior of HDV suggests a network complexity and possible involvement of genetic-environmental factors in the evolution and disease outcomes of distinct genotypes. HDV depends on the HBAg partner and is reported together with HDV genotypes as the main determinants of HDV pathogenicity and antiviral efficacy [39].

Given the recent emergence of novel HDV strains and the increased availability of new genomic information on circulating HDV among the human population, we find it necessary to analyze the genetic diversity and thoroughly clarify the underlying mechanisms behind the evolving HDV, to facilitate the HDV control strategies, guide the HDV molecular epidemiology, and promote the HDV clinical care.

## 2. Methods

### 2.1. Phylogenetic Analysis of HDV

All longer (>1200 nt) HDV genomic sequences isolated between 1986 and 2018 available on the NCBI GenBank database were retrieved and refined based on the genome length. Among a total of 513 downloaded HDV strains, 348 full-length genomes (~1678 nt) from twenty-eight countries all over the world, including Africa (Cameroon, Central African Republic, Nigeria, Gabon, Ivory Coast, Republic of the Congo, Ethiopia, Guinea-Bissau, Togo, and Senegal), Europe (Italy, Spain, Germany, and Russia), Middle East (Turkey, Iran, and Israel), North America (USA and Canada), Latin America (Venezuela, Brazil, and Bolivia), Asia (Kyrgyzstan, Pakistan, Vietnam, China Mainland, China Taiwan, and Japan), and Oceana (Kiribati) were involved in our analysis in the final dataset. HDV genome RNAs encompassing the viroid-like ribozyme-harboring region and the delta antigen coding regions were used in the analysis. The phylogenetic analysis was performed using MEGA11 software [40]. Dendrograms were generated using the Maximum Likelihood method and Tamura Nei model [41]. The tree with the highest log-likelihood was shown. HDV strains are identified in a format as [GenBank ID: virus name (country-year of collection-genotype)].

### 2.2. Similarity Analysis

Genomic similarities between the different HDV genotypes were determined using seventeen representative HDV full-length genome sequences, including one full-length genome sequence from each sub-genotype. The genomic similarity plot was carried out using SimPlot ver.3.5.1 [42].

### 2.3. HDV RNA Recombination

To identify and characterise the recombination events, 348 HDV full-length genome sequences (~1678 nt) were aligned and analysed using the RDP4 software package [43]. The recombination events were identified by each of the seven algorithms, including RDP, GENECONV, Bootscan, MaxChi, Chimaera, SiScan, and 3seq embedded in the RDP4 package. The potential recombinants were further characterised using SimPlot and phylogenetic analysis to verify the recombination authenticity. 

## 3. Results

### 3.1. Phylogenetic Analysis of the Full-Length HDV Genome Sequences

**Figure 1:**
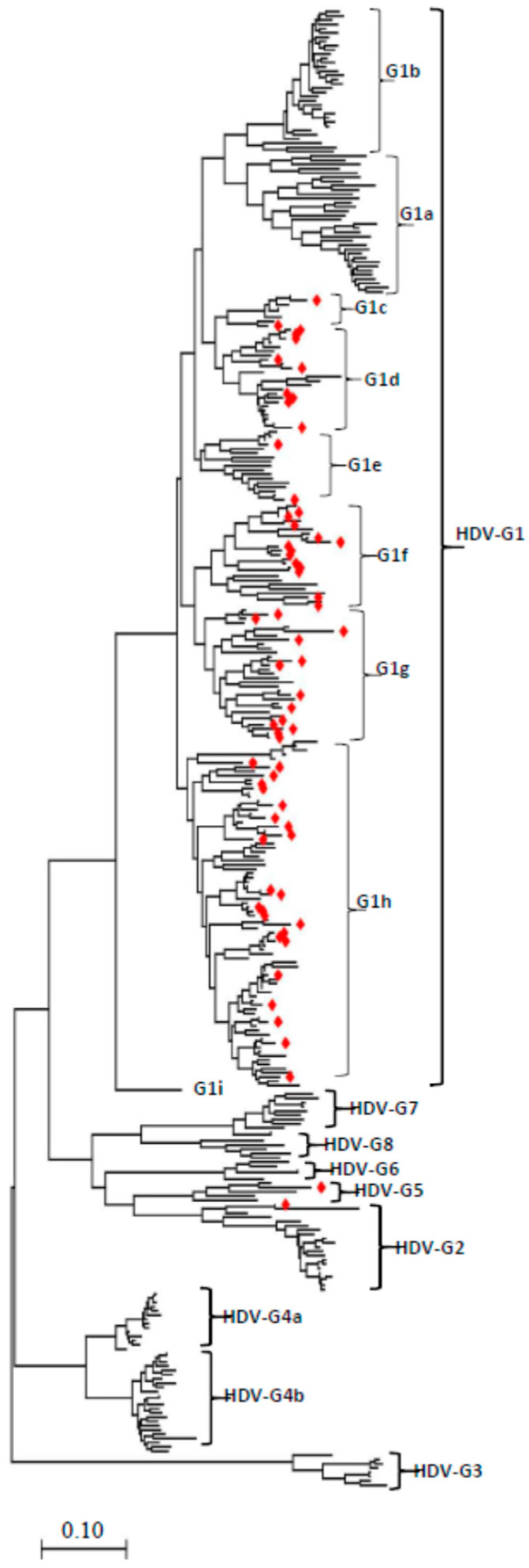
Phylogenetic tree based on 348 human HDV sequences of 1678 nucleotides in length. The tree was constructed using the Maximum Likelihood method in the MEGA-11 software. The Maximum Likelihood method and Tamura Nei model were used to infer the evolutionary history. Initial trees for the heuristic search were automatically obtained by applying Neighbor Join and BioNJ algorithms to a matrix of pairwise distances estimated using the Tamura Nei model and selecting the topology with a superior log-likelihood value. The scale bar refers to a phylogenetic distance of 0.10 nucleotide substitution at each position. The viruses marked in red diamonds represent strains isolated from Kyrgyzstan. Strains are identified in a format as (GenBank ID: virus name (country-year of collection-genotype, and detailed in the Supplementary Figures S1–S6)).

To accurately determine the evolutionary history and connections between HDV genomes, a Maximum Likelihood method was used to perform the phylogenetic analysis of 348 full-length genomic sequences available on the NCBI GenBank database, isolated in different countries all over the world. As indicated in Figure 1 and the Supplementary Figures S1–S6, the genomic region of ~1678 nt was used as a common maximum length of all involved strains. By comparing with the proposed reference strains, the results indicate the existence of eight main HDV genotypes (G1 to G8), and each genotype is shown to be further segregated into two or more sub-genotypes such as G1 segregated into nine sub-genotypes (1a-1i), G2, G3, and G6 into three sub-genotypes (a, b, c), G4, G5, G7, and G8 into two sub-genotypes (a, b). HDV-G3 is shown limited to South America, HDV-G4 is dominant in China (Taiwan) and Japan (Miyako), and HDV-G2 strains (G2a, G2b) are mainly found in Asia as previously reported (Table 1); in addition, a new HDV-G2c sub-genotypes that we found distributed in Kyrgyzstan and Vietnam (Figure 1, Supplementary Figures S1–S6, Table 1). Interestingly, seven new sub-genotypes(G1c–G1i) in HDV-G1 were found, in which most of the HDV strains originating from Kyrgyzstan were sorted (Figure 1, Table 1). HDV G5 strains are mainly found in countries of Africa (Figure 1, Table 1).

**Figure 2:**
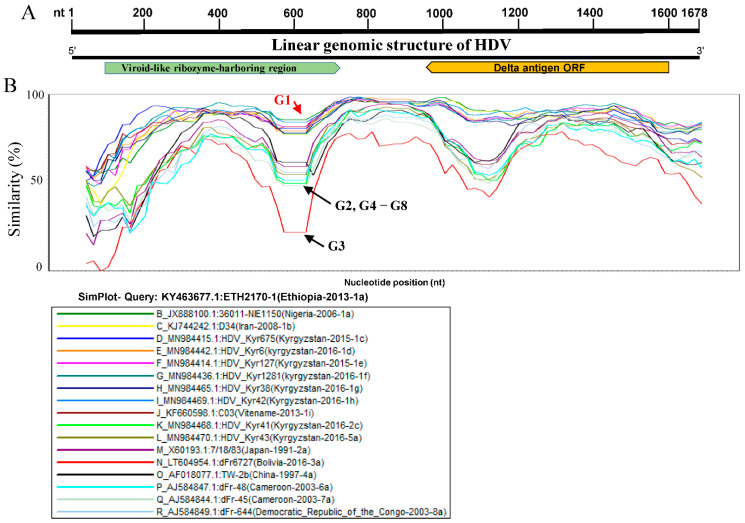
Simplot graph of human HDV genomes. (**A**) Linear Genomic structure of human HDV nucleotide sequences (1678 nt), including the viroid-like ribozyme-harboring region (Rz) and delta antigen ORF. The arrow of delta antigen ORF indicates the direction of translation. (**B**) Comparison of seventeen worldwide representative HDV strains from each sub-genotype in the phylogenetic tree in Figure 1 using similarity analysis. The genomic similarity plot was carried out using SimPlot ver.3.5.1. The X-axes show the nucleotide similarity percentage, and the Y-axes show the nucleotide position. The similarity is presented in percentage (%). The genome of virus ETH2170-1 (GenBank ID: KY463677.1; Ethiopia 2013 1a) was used as SimPlot Query.

To increase the stringency and reliability of our phylogenetic tree findings, we proceed with a similarity analysis, comparing the genome of ETH2170-1 (GenBank ID: KY463677.1, Ethiopia, 2013, HDV1a) belonging to sub-genotypes G1a to seventeen representative HDV full-length sequences using SimPlot analysis. As shown in Figure 2, the viroid-like ribozyme-harboring region (Rz) and the delta antigen ORF region (Figure 2A) exhibited a low similarity percentage (Figure 2), where G3 is shown distant and revealed the lowest level < 25%, [G2, G4–8] revealed ~50%, and G1 strains exhibited >80% of similarity. The two genomic regions revealed a great similarity between HDV-G2 and G4–8 strains (grouped together) and also between the different HDV-G1 sub-genotypes. The results in Figure 2 exhibit that HDV representative viruses fall into distinct groups, indicating an obvious divergence between HDV sub-genotypes (Figure 2). Consistent with the phylogenetic analysis findings, these results suggest that the defined HDV genotypes and sub-genotypes are distinctly shown and highly specific (Figure 2B).

### 3.2. Kyrgyzstan in the Worldwide HDV Genotype Distribution

Curiously, our phylogenetic tree revealed a wide genotype and sub-genotype of Kyrgyzstan HDV strains isolated between 2015 and 2016 (Figure 1, Supplementary Figures S1–S6, Table 1). Kyrgyzstan, a country in Central Asia, has shown the largest spread of HDV genotypes. Kyrgyzstan HDV strains are found largely distributed in HDV-G1 and are classified as HDV-G1 sub-genotypes (c–h) (Figure 1, Supplementary Figures S1–S6, Table 1). An HDV-G5 genotype previously restricted to Africa [32] was also identified in our analysis in Kyrgyzstan (HDV kyr43, GenBank ID: MN 984470.1; 2016), classified as HDV-G5a. Furthermore, our phylogenetic trees identified a novel HDV-G2c sub-genotype that has newly arisen during the HDV evolution, encompassing a Kyrgyzstan strain HDV Kyr41 (GenBank ID: MN984470.1; 2016) with another strain from Vietnam (Supplementary Figure S5, Figure 1).

### 3.3. HDV Inter-Genotype Naturally Occurring Recombinant

To explore the underlying mechanisms and assess the relative contribution of the recombination to the high genetic heterogeneity and dissimilarity observed among HDVs, we performed a recombination analysis of 348 HDV full-length genomes (~1678 nt) using a series of seven algorithms (RDP, Geneconv, BootScan, MaxChi, Chimaera, SiScan, and 3Seq) implemented in the recombination detection program 4 (RDP4) [43]. The recombination analysis detected forty-eight recombination events occurring between 1998 and 2018 (Table 2). So far, we have found twenty-nine novel recombinants isolated from Kyrgyzstan. Moreover, Kyrgyzstan strains recombine in three events with strains from Africa (Togo, Cameroon) (Event 16, 17, and 23), in six events with strains from Europe (Spain, Germany, Russia) (Event 6, 21, 28, 29, 31, and 36), in thirteen events with strains from Asia (China, Vietnam, Kyrgyzstan) (Event 4, 5, 9, 12, 14, 18, 20, 22, 24, 33, 38, 40, and 46), and in five recombination events with strains from the Middle East (Israel, Iran) (Event 7, 8, 10, 13, and 19) (Table 2). Our analysis identified a unique intergenotype recombination Event: DFr2600 (GenBank ID: AM183326) (Event 46). Phylogenetically, the strain DFr2600 in our tree is assigned to G5b and is a recombinant type 2c/5a. DFr2600 intergenotype recombinant strain resulted from recombination between HDV_Kyr43 (GenBank ID: MN984470, G5a) and HDV_Kyr41 (GenBank ID: MN984468. G2c) as major and minor parental strains from Kyrgyzstan, respectively (Table 2). Previously, the HDV-G5 was reported to be restricted to Africa; however, our analysis identified, for the first time, an intercontinental inter-genotype HDV-G5 recombinant strain resulting from a circulating strain in Kyrgyzstan (HDV_Kyr43) that is also involved in the HDV-G5 recombination event 23 as a minor parent. Therefore, we speculate the emergence of a new recombinant lineage (HDV-G5b), and more detailed molecular epidemiological studies are needed to better understand the underlying mechanisms.

### 3.4. HDV Inter-Sub-Genotype Naturally Occurring Recombinants

Our recombination analysis identified thirty-six inter sub-genotype recombinant events (Table 2), in all, thirty-four of which occurred within HDV-G1. Importantly, the quasi-totality of HDV-G1 recombinant strains isolated between 2015 and 2018 was from Kyrgyzstan or at least involved one parental strain from Kyrgyzstan (Table 2). The Kyrgyzstan recombinant strain DFr2600 (G5b) (Event 46) is found to be also implicated as a major parental strain in the recombination Event 23: DFr2703 (GenBank ID: AM183328.1). In Event 23, the DFr2600 strain recombined with HDV_Kyr43 (GenBank ID: MN984470.1) from Kyrgyzstan (Table 2), indicating that DFr2600 can probably recombine with more than two genetically close strains. In addition, we identified a recombination event type 4b/4a (Event 26): JA-M36 (GenBank ID: AB118845.1). The recombinant JA-M36 has already been described previously [44]; however, in our report, for the first time, JA-M36 (HDV-G4a) is revealed to result from recombination between the JA-M11 strain (GenBank ID: AB118826.1) and TWD62*16 (GenBank ID: AY648952.1), from Miyako (Japan) and Taiwan (China), respectively (Table 2).

In line with similarity analysis results and based on the phylogenetic trees, we suggest that until now, the recombination in HDVs genetic material can occur only within the same genotypes, e.g., HDV-G1, HDV-G4, HDV-G5, or between strains that are genetically closer enough such as [HDV-G2c and HDV-G5]. Moreover, Kyrgyzstan strains are largely involved in the extensive recombination events between isolates from different countries and continents, hence in the sustainable HDV evolution (Table 2). Altogether, we suggest a complex HDVs replicative mechanism, where the recombination can occur only between the highly similar HDVs genomes. 

### 3.5. Identification of the Breakpoints for the HDV Recombinants

**Figure 3:**
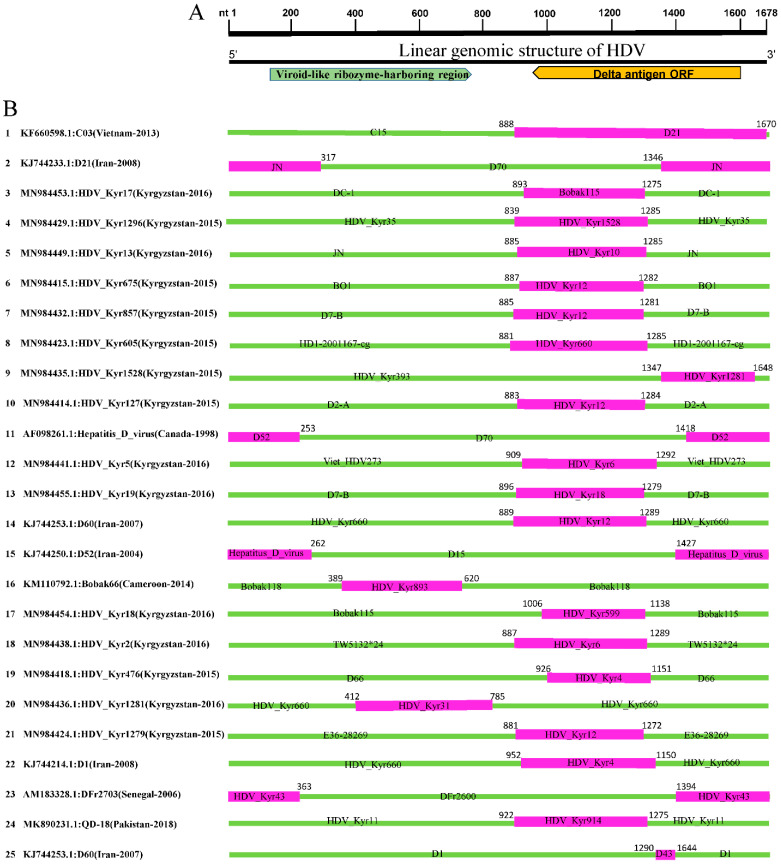
Recombination map across the human HDV genomes for recombination events 1–25. (**A**) Linear genomic structure of HDV full-length sequences (1678 nt). (**B**) Diagram indicating natural recombination events 1–25 detected using the RDP4 software. The numbers on the left indicate the serial number of recombination events. Regions of the major and minor parental sequences are indicated by the green and pink blocks, respectively. Numbers on the top of the blocks indicate the nucleotide positions of breakpoints relative to the full-length genome sequence of the corresponding recombinant viruses.

**Figure 4:**
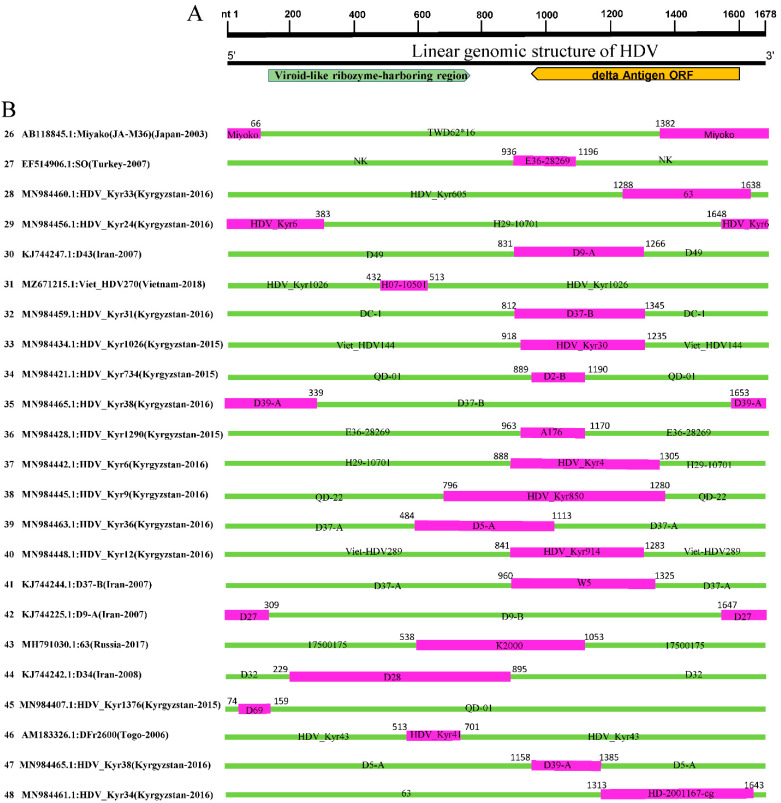
Recombination map across the human HDV genomes for recombination events 26–48. (**A**) Linear genomic structure of HDV full-length sequences (1678 nt). (**B**) Diagram indicating natural recombination events 26–48 detected using the RDP4 software. The numbers on the left indicate the serial number of recombination events. Regions of the major and minor parental sequences are indicated by the green and pink blocks, respectively. Numbers on the top of the blocks indicate the nucleotide positions of breakpoints relative to the full-length genome sequence of the corresponding recombinant viruses.

We mapped the breakpoints of the identified forty-eight HDV recombinants and observed that recombination occurred mainly at the viroid-like ribozyme-harboring region and the delta antigen ORF region (Figure 3 and Figure 4). Five recombination events occurred within the viroid-like ribozyme-harboring region, including Event 16, 20, 31, 44, and 46, with beginning breakpoints at (nt 389, 412, 432, 229, 513) and end breakpoints at (nt 620, 785, 513, 895, 701), respectively. In Events (2, 11, 15, 23, 26, 29, 35, and 42), the recombination beginning and end breakpoints are shown at two different regions of the genome: the viroid-like ribozyme-harboring region and delta antigen ORF coding region, respectively. The rest of the recombinants’ breakpoints are located at similar positions, indicating a hotspot of recombination in HDVs (HDV-G1), with beginning and end breakpoints at or around the delta antigen ORF region (Figure 3 and Figure 4).

### 3.6. Verification of the Identified HDV Recombination Events

**Figure 5:**
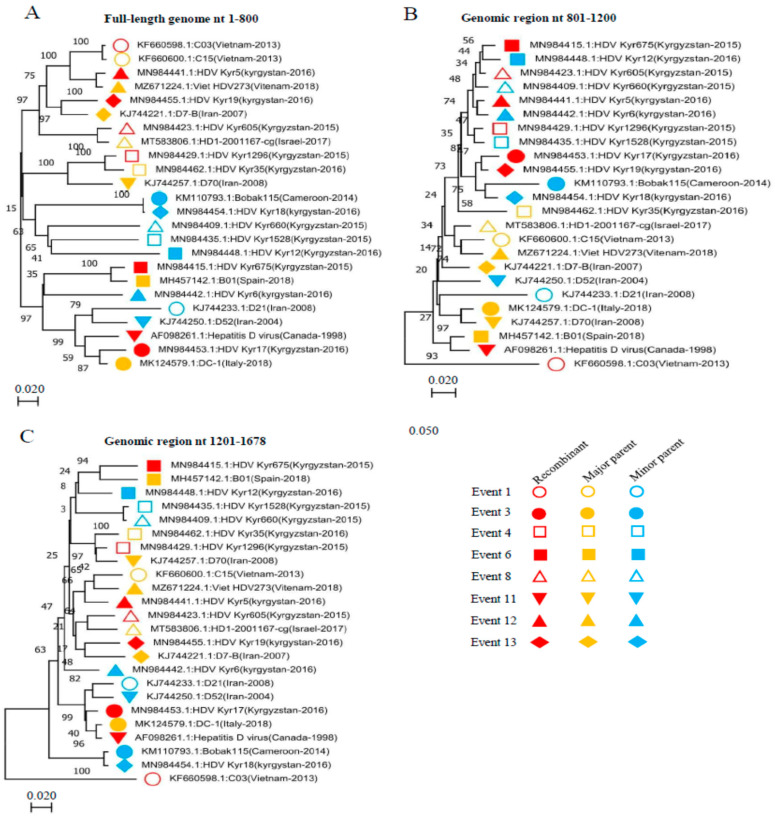
Phylogenetic trees based on three different HDV-RNA genomic regions. (**A**) nt 1–800. (**B**) nt 801–1200. (**C**) nt 1021–1678. Twenty-four HDV strains involved in the recombination events (Event 1, 3, 4, 6, 8, 11, 12, and 13), as an example, were analysed using phylogenetic trees to further confirm the recombination events. The recombinants, minor and major parents, are indicated in red, blue, and yellow colours, respectively.

We further substantiated the putative recombinant events by constructing phylogenetic trees based on three different genomic regions (Figure 5). The recombinant, major, and minor parental sequences are labeled with symbols with three different colours: red, yellow, and blue, respectively. As shown in Figure 5, the recombinant strain in Event 1 (GenBank ID: KF660598.1) is genetically closer to the major parent C15 (GenBank ID: KF660600.1) in the nt 1–800 genomic region-based phylogenetic tree (Figure 5A) but became closer to the minor parent D21 (GenBank ID: KJ744233.1) in the nt 1201–1678 genomic region-based phylogenetic tree (Figure 5C). In Event 3, the recombinant strain (GenBank ID: MN984453.1) is genetically closer to the major parent DC-1 (GenBank ID: MK124579.1) in the nt 1–800 (Figure 5A) and the nt 1201–1678 (Figure 5C) genomic region-based phylogenetic trees but became closer to the minor parent Bobak115 (GenBank ID: KM110793.1) in the nt 801–1200 genomic region-based phylogenetic tree (Figure 5B). Similarly, in Event 4, the recombinant strain (GenBank ID: MN984429.1) is genetically closer to the major parent HDV_Kyr35 (GenBank ID: MN984462.1) in the nt 1–800 (Figure 5A) and nt 1201–1678 (Figure 5B) genomic region-based phylogenetic trees but became closer to the minor parent Kyr1528 (GenBank ID: MN984435.1) in the nt 801–1200 genomic region-based phylogenetic tree (Figure 5B). Similar results are seen with the rest of the recombinant events. Phylogenetic tree results are congruent with the recombination analysis findings, indicating that the detected recombinants resulted from real natural events.

## 4. Discussion

**Figure 6:**
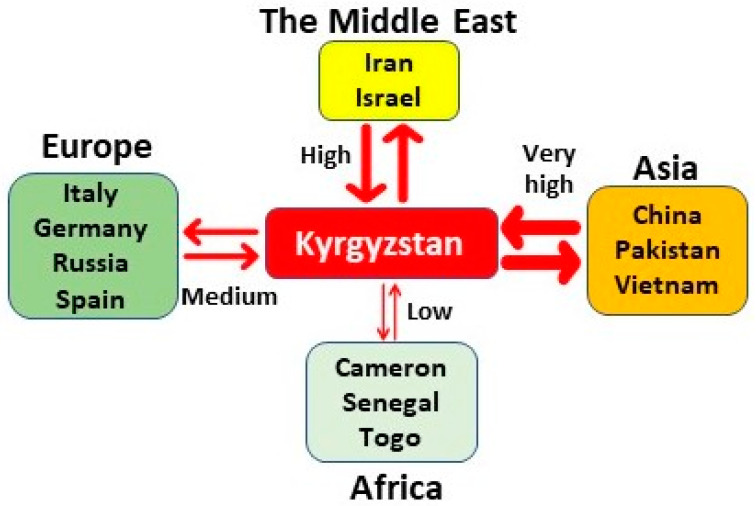
Cartoon linking Kyrgyzstan and the worldwide countries involved in the natural recombination events of HDV. The size of arrows indicates the recombination rate of Kyrgyzstan strains after 2014 associated with geographically distinct areas. The colours Orange, Yellow, Green, and Light Green correspond to very high, high, medium, and low detected recombination rates, respectively.

Recombination in negative-sense single-stranded RNA viruses has been reported to be less frequent due to the genomic structure, the need for a template switch, and limited association of the viral genomic RNA in the RNP complex [45]. Natural recombination in HDV was reported for the first time from a natural mixed-genotypes infected patient (HDV I and IIb) in 2004 and also shown to occur in RNA co-transfection of cultured cells (Wang and Chao, 2005). Two decades later, from the first description, the role of natural recombination in HDV heterogeneity has yet to be systemically explored. In this report, we identified a considerable rate of recombination between HDV full-length genomes, where strains from Kyrgyzstan were extensively involved (Figure 3 and Figure 4; Table 2). Overall, we found forty-eight recombination events out of 348 analysed full-length sequences (~1678 nt), twenty-nine of which are isolated from Kyrgyzstan. Viruses identified in all other continents, including Africa, Europe, the Middle East, and Asia, have been found involved in the recombination of Kyrgyzstan HDVs, indicating the geographic character and the importance of recombination in HDV evolution and propagation (Figure 6).

Furthermore, this report describes a novel unique inter-genotype recombination type 2c/5a and thirty-six inter sub-genotype recombination events, thirty-four of which belong to HDV-G1 type 1d/1h (Table 1). Two of the detected recombinants have been previously reported: JA-M36 (Miyako, Japan) and C03 (Vietnam) (Table 1) [44,46]. The breakpoint of the novel 2c/5a recombinant is identified to be at the (513–701 nt) position corresponding to the viroid-like ribozyme-harboring region, while for the remaining recombinants, the breakpoints were mapped occurring throughout the whole HDV genome, especially near or at the delta antigen coding region (Figure 3 and Figure 4).

It has been documented that Asia and Africa are the largest HBV reservoirs continents and thus at high risk of undergoing an exaggerated burden of HDV infection [47]. Consistently, our data revealed that the largest HDV genotype diversity is currently determined in Kyrgyzstan, which we also identified as the new land of the most HDV recombination events occurring after 2014 (Figure 6). Kyrgyzstan is one of the WHO’s viral hepatitis focus countries; however, the country’s situation in terms of HBV and HCV prevalence is unknown due to the absence of population-based nationally representative studies [48]. A study conducted on thirty plasmas collected from chronic HBV and HDV patients across Kyrgyzstan [49] revealed that by using phylogenetic analysis that all of the included patients were HBV genotype D and dominated by HBV subtype D1 (73.34%). HDV genotype I was also identified with a highly variable delta antigen coding region [49]. Importantly, this report suggested the occurrence of several independent genetic drifts in HDV strains into the territory of Kyrgyzstan with a high speed of HDV evolution in the region [49]. Congruently with Semenov A.V. et al. report, our results demonstrate that Kyrgyzstan prevails with the highest number of HDV-G1 emerging recombinants. Therefore, we suggest Kyrgyzstan as the new epicenter of HDV infection in Central Asia (based on the available HDV genomes) (Figure 6). Furthermore, a hypothesis can be formulated linking the great recombination potential of Kyrgyzstan HDVs and their rapid dispersal to the fact that Central Asia has been characterised by the movement of people throughout history and the high internal and external migration of Kyrgyz people. The economy of Kyrgyzstan sorely depends on the labour migration, where approximately 50,000 Kyrgyz every year leave Kyrgyzstan to work abroad, mostly in the Russian Federation, Turkey, and United Arab Emirates, being exposed to human trafficking and abuse [50]. Human exploitation, injecting drugs, and substance use disorder might be behind the diversity of the Kyrgyzstan HDVs strains, which need more exploration.

Moreover, Kiesslich et al. conducted a study to evaluate the clinical impact of HBV genotypes in the case of HBV/HDV co-infection [51]. The study involved 190 patients, 140 of whom had chronic HBV mono-infection and 50 had chronic HBV-HDV co-infection [51]. Interestingly, the HDV/HBV genotype F showed a greater alanine aminotransferase (ALT) level than HBV genotype F mono-infection, indicating that dual infection and the viral genotype led to greater liver disease severity and inflammatory potential [51]. The HDV viral load was observed to be lower in the case of HBV genotype A compared to HBV genotype D [51]. Therefore, the association between the HBV/HDV co-infection, the genotypes of the two viruses, the viremia, and clinical outcomes needs further investigations with consideration of the emerging Kyrgyzstan HDV strains, which would be of great significance and may increase the understanding of the HDV molecular virology.

Previously, Lin et al. [44] reported a novel natural recombinant JA-M36 in which the breakpoints were located at 68 and 1392 nt positions, with potential major (JA-M33) and minor (JA-M11) parental sequences isolated in Miyako Island [44]. Similarly, the JA-M36 strain in our report was identified as recombinant (Event 26) with beginning and end breakpoints at nt 1382 and nt 66, respectively. However, JA-M36 in our analysis is assigned to G4a and found to result from recombination between the minor Miyako (JA-M11) and major TWD62*16 (Taiwan-2004) parents, indicating that the JA-M36 strain may result from different origins, which may be related to people migration between the two close areas. Moreover, Sy et al. identified a natural inter-genotype HDV recombination between HDV-1 and HDV-2 and yielded the C03 Vietnam strain [46]. The sub-genomic analyses of the HDAg domain coding region (nt 1013–1600) exhibited that the C03 Vietnam isolates belonged to the HDV-2 clade, while the full-length genome analysis indicated that C03 clustered into HDV-1, suggesting inter-genotypic recombination [46]. The crossover region was identified between nt 800 and nt 950; however, the comparison of C03 sequences of HDV-1 (GenBank ID: M92448) and HDV-2 (GenBank ID: AF425645) fragments ranging between nt 673 and nt 1118 identified one breakpoint at nt 908 corresponding to the downstream of riboszyme-containing region [46]. These findings have been re-evaluated by Lin et al. [44], exhibiting that C03 is an inter-genotypic HDV-1/HDV-2 recombinant, which shared a high sequence homology of >99% with C15 strain and 97.8% with C6 strain [44]. Here, we identified C03 Vietnam as an HDV-G1 inter-sub-genotypic recombinant type (1d/1h) between strains from two different countries: D21 (Iran: 1i) and C15 (Vietnam: 1h) (Figure 3, Table 2). Our findings suggest that HDV genetic recombination during the RNA replication involving delta antigen coding sequences and the viroid-like ribozyme-harboring region (Rz) is one of the viral heterogeneity mechanisms that stipulate the complexity of HDV genetic networks and interactions. These results will help promote the viral hepatitis prevention and control strategies; however, the importance of the Kyrgyzstan HDVs recombination in the evolution of HDV remains uncertain and needs to be confirmed in a patient coinfected with more than one HDV strain.

Despite the vaccination programs against the Hepatitis B virus that have reversed the epidemiology of the HDV infection, hepatitis D, clinically associated with a poor prognosis, remains a global health issue with great medical importance [52]. HDV is highly prevalent in specific endemic areas, where the geographical distribution is linked to discrepancies in the molecular genotypes [5]. HDV infection has been reported to be highly prevalent in Asia, Europe, Latin America, and sub-Saharan Africa [4,5]; however, accurate estimates of the regional distribution and the correlation of HDV disease manifestations with genotypes’ diversity and distribution are still vague and not fully clear. Eight HDV genotypes have been recently reported and are generally acknowledged (HDV-1 to HDV-8) [32]. Herein, we used the Maximum Likelihood (ML) approach to update the HDV genotyping by including the newly available genetic information and using full-length genomes with longer sequences (~1678 nt in length) isolated between 1986 and 2018. Phylogenetically, our results corroborate the current classification system, revealing eight main HDV genotypes [32], but expand with the addition of sub-genotypes of HDV1 (1e–i) and HDV2 (2c).

Recently, Spaan et al. [53], in a retrospective study conducted on 107 patients from different origins (64% of African origin and 17% of European origin), reported a distinct clinical behavior of HDV-1 compared to HDV-5 [53]. Patients genotyped HDV-5, mostly of an African origin, showed more favorable liver disease progression with less hepatic decompensation episodes than those genotyped HDV-1 (from European origin, 56%) [53]. Furthermore, patients with HDV-5 responded more appropriately to Pegylated interferon (peg-IFN) treatment than those with HDV-1 [53]. Le Gal et al. have reported that patients infected with [European/Asian] HDV-1 strains showed an occurrence of cirrhosis twice more often than patients infected with African HDV-1 strains [32]. These findings, revealing the distinct clinical behavior of HDV genotypes among individuals, could be linked to the remarkable HDV-G1 diversity and extensive recombination patterns, reflecting distinct and complex replication mechanisms of the HDVs RNA. Our phylogenetic trees corroborate taxa classification, showing that HDV-G3 is restricted to South America, HDV-G4 to Asia (Japan, China), and HDV-G6 to G8 are limited to Africa. However, HDV-5, which was reported restricted to Africa [32], is found in our analysis circulating in Asia (Kyrgyzstan). An extension of the HDV-G2 sub-genotypes with additional HDV-G2c is also identified in our results (distributed in Kyrgyzstan and Vietnam), in contrast to the adopted HDV classification system, grouping HDV-2 into only two sub-genotypes (HDV-2a and 2b). Thus, we recommend more epidemiological and molecular investigations considering the demographic data and sociocultural and traditional beliefs in studying the propagation of diverse HDV genotypes and their clinical impacts.

## 5. Conclusions

In summary, we suggest that the diversity and wide distribution of Kyrgyzstan HDVs may be directed by the migration flow and people movement, leading to the emergence of new complex HDV lineages. The limited distribution of some genotypes to particular areas may be linked to indigenous populations and sociocultural traditions and beliefs. Therefore, more epidemiological studies taking into account the geographical distribution of HBV/HDV and sociocultural differences would be of great benefit to clarify the correlation between the worldwide Kyrgyzstan strains dispersal and the HDV clinical outcomes; hence promoting the effectiveness of prevention, clinical care, and public health management of chronic hepatitis D.

## Figures and Tables

**Table 1:** Geographic distribution of HDV genotypes and sub-genotypes.

HDV Genotype		Africa	Middle East	Europe	Asia	America	Oceania
G1	a	Cameroon, Ethiopia, Nigeria, Somalia	Iran		Pakistan		
	b		Iran	Germany			Kiribati
	c		Iran, Israel	Italy, Spain	Kyrgyzstan	USA	
	d	The Central African Republic	Iran	Germany, Italy, Spain	China, Kyrgyzstan	Canada, USA	
	e		Iran, Israel		Kyrgyzstan, Pakistan		
	f	Cameroon, Ethiopia, Nigeria,	Iran,	Germany	Kyrgyzstan, Pakistan		
	g		Iran, Israel		Kyrgyzstan		
	h		Iran, Israel, Turkey	Germany, Italy, Russia	China, Japan, Kyrgyzstan, Vietnam	Brazil, USA	
	i				Vietnam		
G2	a				China (Taiwan), Japan, Vietnam		
	b			Russia	China (Taiwan)		
	c				Kyrgyzstan, Vietnam		
G3	a					Bolivia	
	b					Brazil, Venezuela	
	c					Brazil	
G4	a				China, China (Taiwan), Japan		
	b				Japan		
G5	a	Nigeria, Senegal			Kyrgyzstan		
	b	Guinea-Bissau, Togo					
G6	a	Cameroon					
	b	Nigeria					
	c	The Central African Republic,					
G7	a	Cameroon					
	b	Cameroon					
G8	a	Democratic Republic of the Congo, Cote d’Ivoire,					
	b	Democratic Republic of the Congo, Gabon, Senegal					

**Table 2:** The potential recombination events in the genome of HDVs. R, RDP; G, GENECONV; B, BootScan; M, MaxChi; C, Chimaera; S, SiScan; T, 3seq. +, verified; -, not verified. * The major or minor parent may be the actual recombinant due to the possibility of misidentification.

Recombination Event Serial Number	Recombinant		Minor Parent		Major Parent		Detection Methods						
	GenBank ID: Virus Name (Country-Year)	Genotype	GenBank ID: Virus Name (Country-Year)	Genotype	GenBank ID: Virus Name (Country-Year)	Genotype	R	G	B	M	C	S	T
1	KF660598.1:C03 (Vietnam-2013)	**G1i**	KJ744233.1:D21 (Iran-2008)	**G1d**	KF660600.1:C15 (Vietnam-2013)	**G1h**	+	+	+	+	+	-	+
2	* KJ744233.1:D21 (Iran-2008)	**G1d**	HM046802.1:JN (China-2010)	**G1d**	KJ744257.1:D70 (Iran-2008)	**G1g**	+	+	+	+	-	+	+
3	MN984453.1:HDV_Kyr17 (Kyrgyzstan-2016)	**G1d**	KM110793.1:Bobak115 (Cameroon-2014)	**G1f**	MK124579.1:DC-1 (Italy-2018)	**G1d**	+	+	+	+	+	+	+
4	* MN984429.1:HDV_Kyr1296 (Kyrgyzstan-2015)	**G1g**	MN984435.1:HDV_Kyr1528 (Kyrgyzstan-2015)	**G1f**	MN984462.1:HDV_Kyr35 (Kyrgyzstan-2016)	**G1g**	+	+	+	+	+	+	+
5	MN984449.1:HDV_Kyr13 (Kyrgyzstan-2016)	**G1d**	MN984446.1:HDV_Kyr10 (Kyrgyzstan-2016)	**G1g**	HM046802.1:JN (China-2010)	**G1d**	+	+	+	+	+	+	+
6	MN984415.1:HDV_Kyr675 (Kyrgyzstan-2015)	**G1c**	MN984448.1:HDV_Kyr12 (kygyzstan-2016)	**G1f**	MH457142.1:B01 (Spain-2018)	**G1c**	+	+	+	+	+	+	+
7	MN984432.1:HDV_Kyr857 (Kyrgyzstan-2015)	**G1h**	MN984448.1:HDV_Kyr12 (kygyzstan-2016)	**G1f**	KJ744221.1:D7-B (Iran-2007)	**G1h**	+	+	+	+	+	+	+
8	MN984423.1:HDV_Kyr605 (Kyrgyzstan-2015)	**G1h**	MN984409.1:HDV_Kyr660 (Kyrgyzstan-2015)	**G1f**	MT583806.1:HD1-2001167-cg (Israel-2017)	**G1h**	+	+	+	+	+	+	+
9	* MN984435.1:HDV_Kyr1528 (Kyrgyzstan-2015)	**G1f**	MN984436.1:HDV_Kyr1281 (Kyrgyzstan-2016)	**G1f**	MN984416.1:HDV_Kyr393 (Kyrgyzstan-2015)	**G1f**	+	+	+	+	+	+	+
10	MN984414.1:HDV_Kyr127 (Kyrgyzstan-2015)	**G1e**	MN984448.1:HDV_Kyr12 (Kygyzstan-2016)	**G1f**	KJ744215.1:D2-A (Iran-2006)	**G1e**	+	+	-	+	+	+	+
11	AF098261.1:Hepatitis_D_virus (Canada-1998)	**G1d**	KJ744250.1:D52 (Iran-2004)	**G1d**	KJ744257.1:D70 (Iran-2008)	**G1g**	+	+	+	+	+	+	+
12	MN984441.1:HDV_Kyr5 (Kyrgyzstan-2016)	**G1h**	MN984442.1:HDV_Kyr6 (Kyrgyzstan-2016)	**G1d**	MZ671224.1:Viet_HDV273 (Vietnam-2018)	**G1h**	+	+	+	+	+	+	+
13	* MN984455.1:HDV_Kyr19 (Kyrgyzstan-2016)	**G1h**	MN984454.1:HDV_Kyr18 (Kyrgyzstan-2016)	**G1f**	KJ744221.1:D7-B (Iran-2007)	**G1h**	+	+	+	+	+	+	+
14	* KJ744253.1:D60 (Iran-2007)	**G1f**	MN984448.1:HDV_Kyr12 (Kygyzstan-2016)	**G1f**	MN984409.1:HDV_Kyr660 (Kyrgyzstan-2015)	**G1f**	+	+	+	+	+	+	+
15	* KJ744250.1:D52 (Iran-2004)	**G1d**	AF098261.1:Hepatitis_D_virus (Canada-1998)	**G1d**	KJ744230.1:D15 (Iran-2004)	**G1c**	+	+	+	+	+	+	+
16	KM110792.1:Bobak66 (Cameroon-2014)	**G1a**	MN984427.1:HDV_Kyr893 (Kyrgyzstan-2015)	**G1f**	KM110794.1:Bobak118 (Cameroon-2014)	**G1a**	+	-	+	+	+	-	+
17	* MN984454.1:HDV_Kyr18 (Kyrgyzstan-2016)	**G1f**	MN984412.1:HDV_Kyr599 (Kyrgyzstan-2015)	**G1f**	KM110793.1:Bobak115 (Cameroon-2014)	**G1f**	+	+	-	-	-	+	+
18	MN984438.1:HDV_Kyr2 (Kyrgyzstan-2016)	**G1h**	MN984442.1:HDV_Kyr6 (Kyrgyzstan-2016)	**G1d**	AY648957.1:TW5132*24 (Taiwan-2004)	**G1h**	+	+	+	+	+	+	+
19	* MN984418.1:HDV_Kyr476 (Kyrgyzstan-2015)	**G1g**	MN984440.1:HDV_Kyr4 (Kyrgyzstan-2016)	**G1h**	KJ744255.1:D66 (Iran-2003)	**G1g**	+	+	-	+	-	+	+
20	MN984436.1:HDV_Kyr1281 (Kyrgyzstan-2016)	**G1f**	MN984459.1:HDV_Kyr31 (Kyrgyzstan-2016)	**G1d**	MN984409.1:HDV_Kyr660 (Kyrgyzstan-2015)	**G1f**	+	+	+	+	+	+	+
21	* MN984424.1:HDV_Kyr1279 (Kyrgyzstan-2015)	**G1d**	MN984448.1:HDV_Kyr12 (Kygyzstan-2016)	**G1f**	MH457147.1:E36-28269 (Germany-2018)	**G1d**	+	+	+	+	+	-	+
22	* KJ744214.1:D1 (Iran-2008)	**G1f**	MN984440.1:HDV_Kyr4 (Kyrgyzstan-2016)	**G1h**	MN984409.1:HDV_Kyr660 (Kyrgyzstan-2015)	**G1f**	+	+	-	-	-	-	+
23	* AM183328.1:DFr2703 (Senegal-2006)	**G5a**	MN984470.1:HDV_Kyr43 (Kyrgyzstan-2016)	**G5a**	AM183326.1:DFr2600 (Togo-2006)	**G5b**	+	+	+	+	+	+	+
24	* MK890231.1:QD-18 (Pakistan-2018)	**G1f**	MN984413.1:HDV_Kyr914 (Kyrgyzstan-2015)	**G1g**	MN984447.1:HDV_Kyr11 (Kyrgyzstan-2016)	**G1f**	+	+	-	+	+	-	+
25	* KJ744253.1:D60 (Iran-2007)	**G1f**	KJ744247.1:D43 (Iran-2007)	**G1g**	KJ744214.1:D1 (Iran-2008)	**G1f**	+	+	+	+	+	+	+
26	AB118845.1:Miyako (JA-M36) (Japan-2003)	**G4a**	AB118826.1:Miyako (JA-M11) (Japan-2003)	**G4b**	AY648952.1:TWD62*16 (Taiwan-2004)	**G4a**	+	+	-	+	+	+	+
27	* EF514906.1:SO (Turkey-2007)	**G1h**	MH457147.1:E36-28269 (Germany-2018)	**G1d**	EF514904.1:NK (Turkey-2007)	**G1h**	+	+	+	+	+	+	+
28	* MN984460.1:HDV_Kyr33 (Kyrgyzstan-2016)	**G1h**	MH791030.1:63 (Russia-2017)	**G1h**	MN984423.1:HDV_Kyr605 (Kyrgyzstan-2015)	**G1h**	+	+	+	+	+	+	+
29	* MN984456.1:HDV_Kyr24 (Kyrgyzstan-2016)	**G1d**	MN984442.1:HDV_Kyr6 (Kyrgyzstan-2016)	**G1d**	MH457150.1:H29-10701 (Germany-2018)	**G1d**	+	+	+	+	+	+	+
30	KJ744247.1:D43 (Iran-2007)	**G1g**	KJ744225.1:D9-A (Iran-2007)	**G1g**	KJ744248.1:D49 (Iran-2010)	**G1g**	+	+	+	+	+	-	+
31	* MZ671215.1:Viet_HDV270 (Vietnam-2018)	**G1h**	MH457149.1:H07-10501 (Germany-2018)	**G1b**	MN984434.1:HDV_Kyr1026 (Kyrgyzstan-2015)	**G1h**	+	+	+	+	+	+	+
32	* MN984459.1:HDV_Kyr31 (Kyrgyzstan-2016)	**G1d**	KJ744244.1:D37-B (Iran-2007)	**G1g**	MK124579.1:DC-1 (Italy-2018)	**G1d**	+	+	-	+	+	-	+
33	* MN984434.1:HDV_Kyr1026 (Kyrgyzstan-2015)	**G1h**	MN984458.1:HDV_Kyr30 (Kyrgyzstan-2016)	**G1h**	MZ671212.1:Viet_HDV44 (Vietnam-2018)	**G1h**	+	+	+	+	+	-	+
34	* MN984421.1:HDV_Kyr734 (Kyrgyzstan-2015)	**G1f**	KJ744216.1:D2-B (Iran-2007)	**G1e**	MK890225.1:QD-01 (Pakistan-2018)	**G1f**	+	+	-	+	+	+	+
35	* MN984465.1:HDV_Kyr38 (Kyrgyzstan-2016)	**G1g**	KJ744245.1:D39-A (Iran-2006)	**G1g**	KJ744244.1:D37-B (Iran-2007)	**G1g**	+	-	+	-	-	+	+
36	MN984428.1:HDV_Kyr1290 (Kyrgyzstan-2015)	**G1d**	MG711663.1:A176 (Cameroon-2015)	**G1a**	MH457147.1:E36-28269 (Germany-2018)	**G1d**	+	+	-	+	+	+	+
37	* MN984442.1:HDV_Kyr6 (Kyrgyzstan-2016)	**G1d**	MN984440.1:HDV_Kyr4 (Kyrgyzstan-2016)	**G1h**	MH457150.1:H29-10701 (Germany-2018)	**G1d**	+	+	-	+	+	+	+
38	* MN984445.1:HDV_Kyr9 (Kyrgyzstan-2016)	**G1e**	MN984419.1:HDV_Kyr850 (Kyrgyzstan-2015)	**G1f**	MK890232.1:QD-22 (Pakistan-2018)	**G1e**	+	+	+	+	+	+	+
39	* MN984463.1:HDV_Kyr36 (Kyrgyzstan-2016)	**G1g**	KJ744218.1:D5-A (Iran-2006)	**G1g**	KJ744243.1:D37-A (Iran-2007)	**G1g**	+	+	-	+	+	+	+
40	* MN984448.1:HDV_Kyr12 (Kyrgyzstan-2016)	**G1f**	MN984413.1:HDV_Kyr914 (Kyrgyzstan-2015)	**G1g**	MZ671223.1:Viet_HDV289 (Vietnam-2018)	**G1h**	+	+	+	+	+	+	+
41	* KJ744244.1:D37-B (Iran-2007)	**G1g**	AJ307077.1:W5 (Italy-2001)	**G1d**	KJ744243.1:D37-A (Iran-2007)	**G1g**	+	+	-	+	+	+	+
42	* KJ744225.1:D9-A (Iran-2007)	**G1g**	KJ744237.1:D27 (Iran-2008)	**G1e**	KJ744226.1:D9-B (Iran-2008)	**G1g**	+	-	-	+	+	+	+
43	* MH791030.1:63 (Russia-2017)	**G1h**	MG711712.1:K2000 (Cameroon-2013)	**G1a**	MT649284.1:17500175 (Kiribati-2017)	**G1b**	+	-	-	+	+	+	+
44	* KJ744242.1:D34 (Iran-2008)	**G1b**	KJ744238.1:D28 (Iran-2008)	**G1e**	KJ744240.1:D32 (Iran-2008)	**G1b**	+	-	-	+	+	+	+
45	* MN984407.1:HDV_Kyr1376 (Kyrgyzstan-2015)	**G1f**	KJ744256.1:D69 (Iran-2005)	**G1g**	MK890225.1:QD-01 (Pakistan-2018)	**G1f**	+	+	-	+	+	-	-
46	* AM183326.1:DFr2600 (Togo-2006)	**G5b**	MN984468.1:HDV_Kyr41 (Kyrgyzstan-2016)	**G2c**	MN984470.1:HDV_Kyr43 (Kyrgyzstan-2016)	**G5a**	+	-	+	+	+	+	+
47	* MN984465.1:HDV_Kyr38 (Kyrgyzstan-2016)	**G1g**	KJ744245.1:D39-A (Iran-2006)	**G1g**	KJ744218.1:D5-A (Iran-2006)	**G1g**	+	+	+	-	+	+	+
48	MN984461.1:HDV_Kyr34 (Kyrgyzstan-2016)	**G1h**	MT583806.1:HD1-2001167-cg (Israel-2017)	**G1h**	MH791030.1:63 (Russia-2017)	**G1h**	+	+	-	+	+	-	+

## Data Availability

The data are available online on the National Center for Biotechnology Information (NCBI) website.

## Supplementary Materials

The following supporting information can be downloaded at: https://www.mdpi.com/article/10.3390/v14071467/s1, Figure S1–S6: Phylogenetic tree based on HDV genomes of 1678 nt.
