# Supplementary material for: Global Distribution and Natural Recombination of Hepatitis D Virus: Implication of Kyrgyzstan Emerging HDVs in the Clinical Outcomes
Source: Viruses. 2022 Jul 2;14(7):1467. doi: 10.3390/v14071467 (PMC9323457; doi:10.3390/v14071467)
Supplement: Supplementary file 1 [file viruses-14-01467-s001.zip › viruses-1798093-supplementary.pdf]

Global Distribution and Natural Recombination of Hepatitis D Virus: Implication of Kyrgyzstan Emerging HDVs in the Clinical Outcomes

Amina Nawal Bahoussi[1], Pei-Hua Wang[1], Yan-Yan Guo[1], Nighat Rabbani[1], Changxin Wu[1,2,3], Li Xing[1,2,3]*

Supplementary Figure S1-6, Phylogenetic tree based on HDV genomes of 1678 nt.

Supplementary Figure S1. Phylogenetic tree based on HDV genomes of 1678 nt, including (HDV- G1a and G1b isolates). The tree was constructed using the Maximum Likelihood method in the MEGA-11 software. The Maximum Likelihood method and Tamura Nei model were used to infer the evolutionary history. Initial trees for the heuristic search were automatically obtained by applying Neighbor Join, and BioNJ algorithms to a matrix of pairwise distances estimated using the Tamura Nei model and selecting the topology with a superior log-likelihood value. The scale bar refers to a phylogenetic distance of 0.10 nucleotide substitution at each position. The viruses marked in red represent strains isolated from Kyrgyzstan. Strains are identified in a format as [GenBank ID: virus name (country-year of collection-genotype). Highlighted is the reference HDV for each sub-genotype.

Supplementary Figure S2. Phylogenetic tree based on HDV genomes of 1678 nt, including (HDV-G1c, G1d, G1e and G1f isolates) (Continued).

Supplementary Figure S3. Phylogenetic tree based on HDV genomes of 1678, including (HDV-G1f, G1g, and G1h isolates) (Continued).

Supplementary Figure S4. Phylogenetic tree based on HDV genomes of 1678, including (HDV-G1h and G1i isolates) (Continued).

Supplementary Figure S5. Phylogenetic tree based on HDV genomes of 1678, including (HDV-G7, G8, G6, G5, and G2 isolates) (Continued).

Supplementary Figure S6. Phylogenetic tree based on HDV genomes of 1678nt including (HDV-G3 and G4 isolates) (Continued).

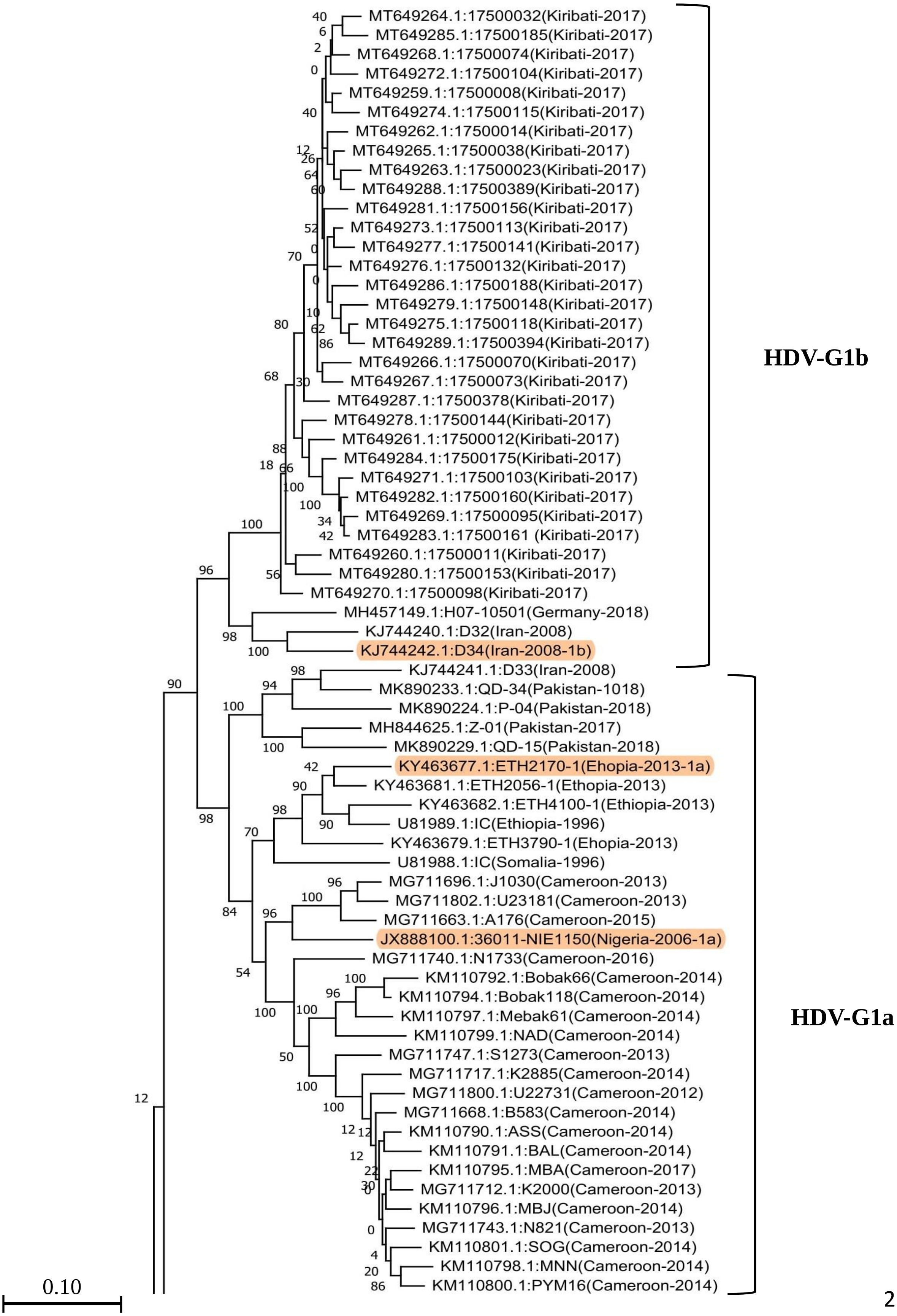

Supplementary Figure S1

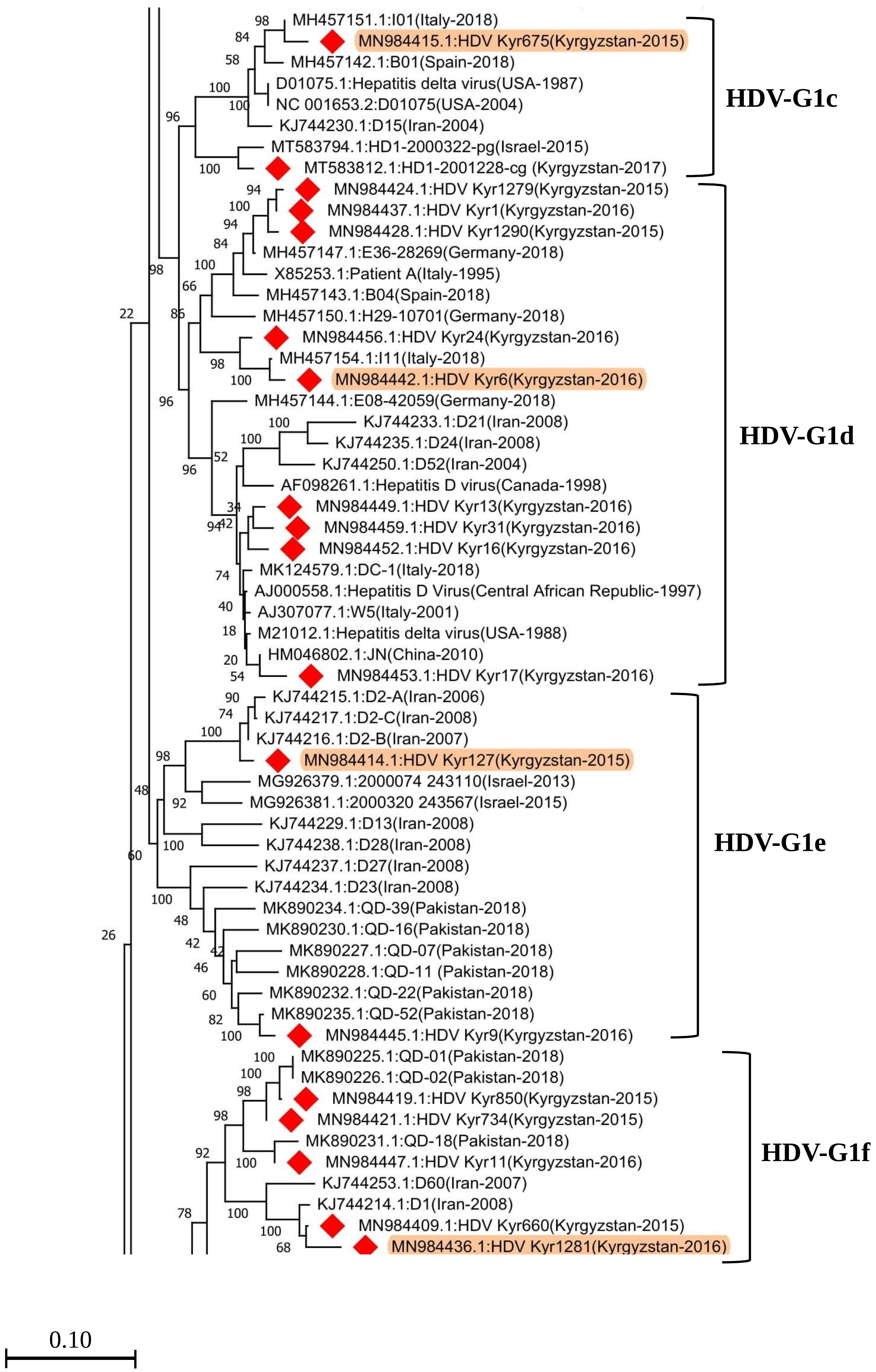

Supplementary Figure S2

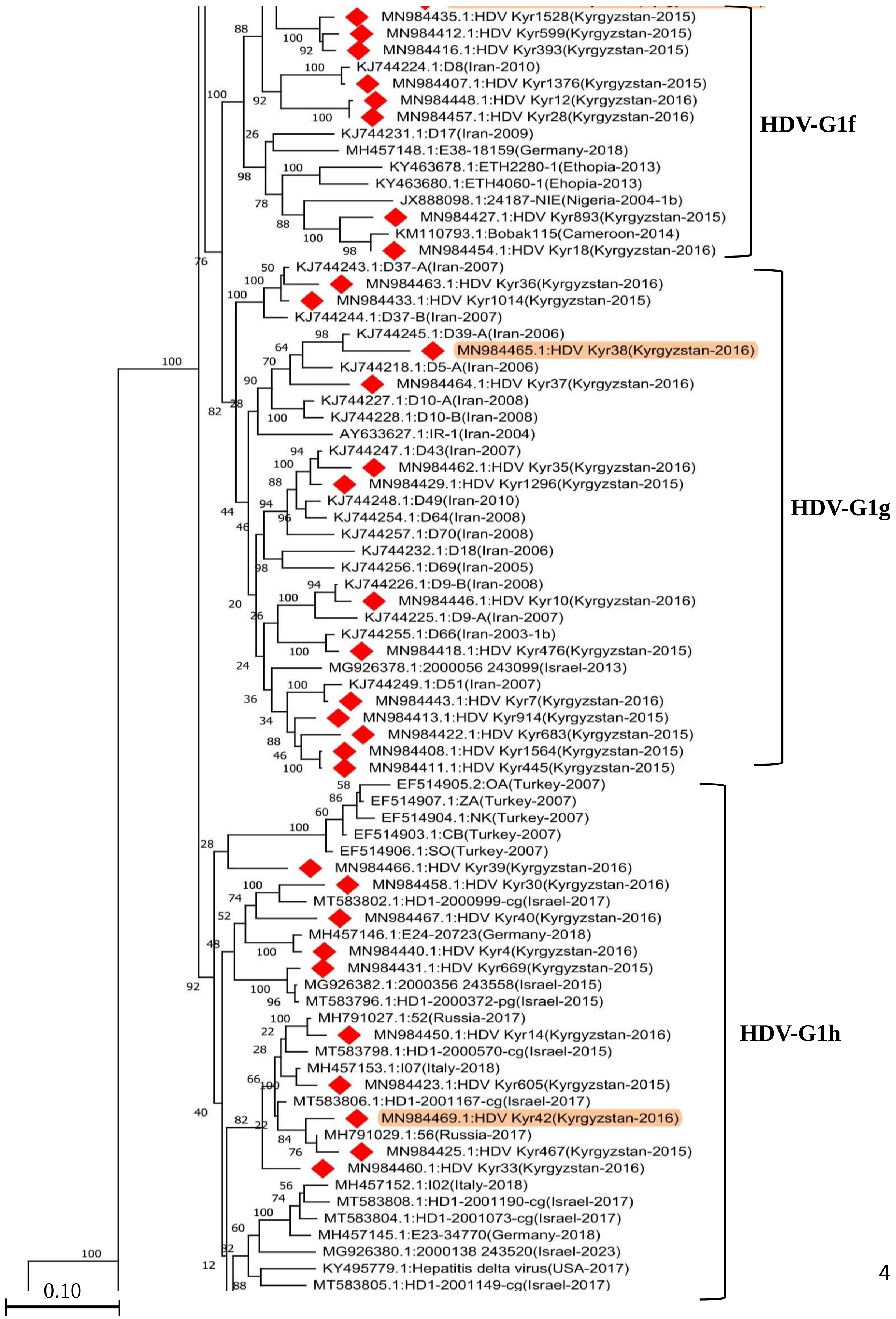

Supplementary Figure S3

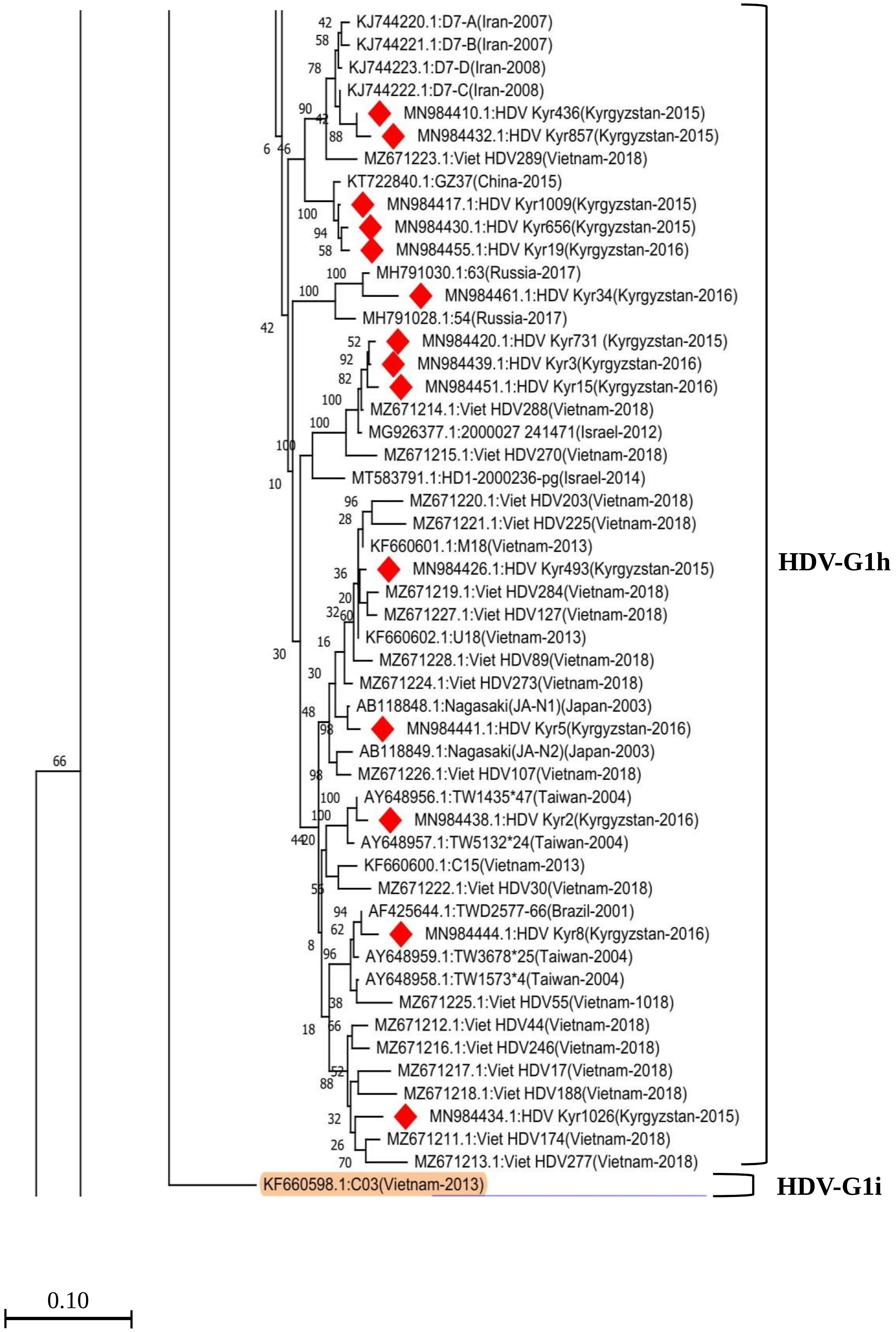

Supplementary Figure S4

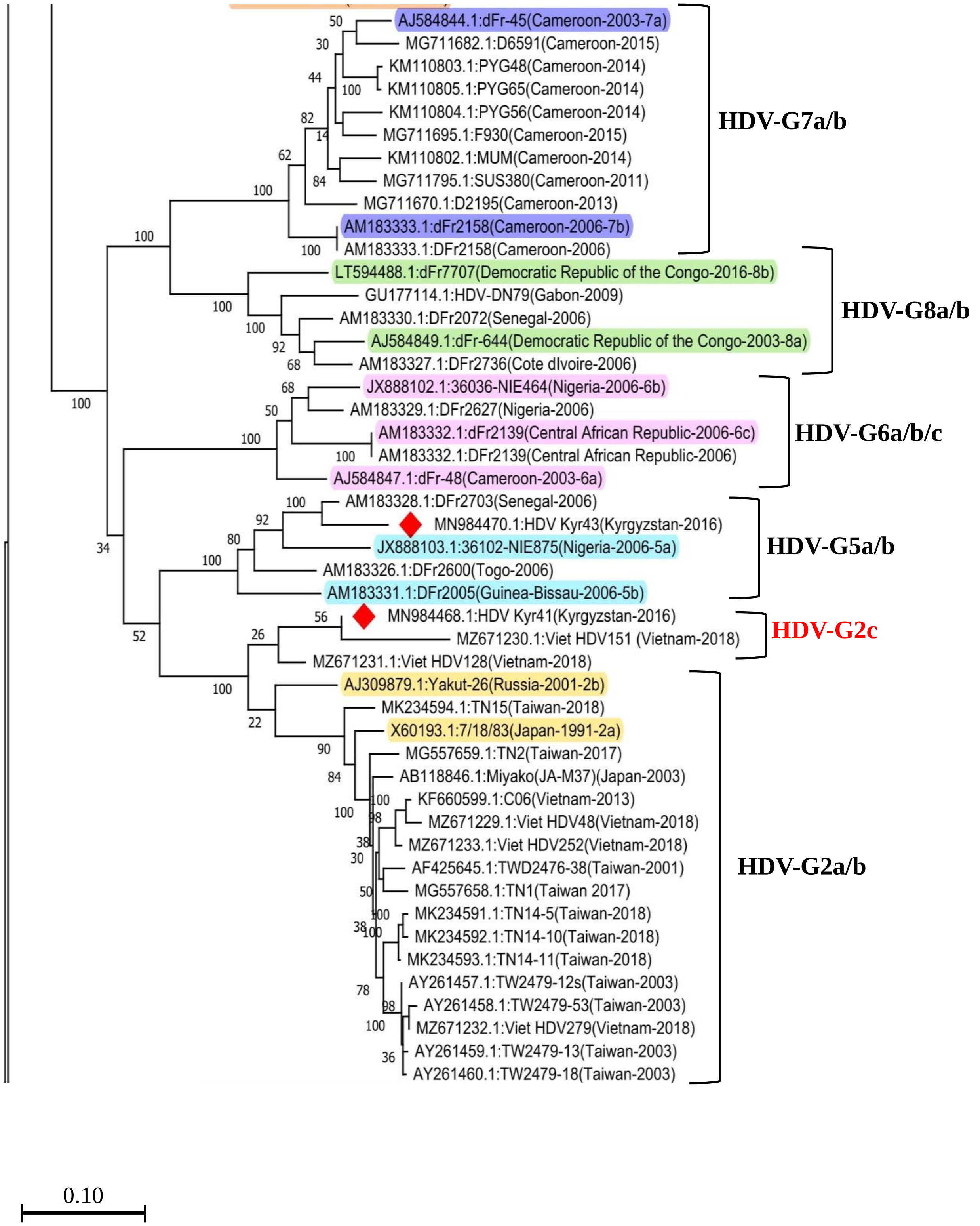

Supplementary Figure S5

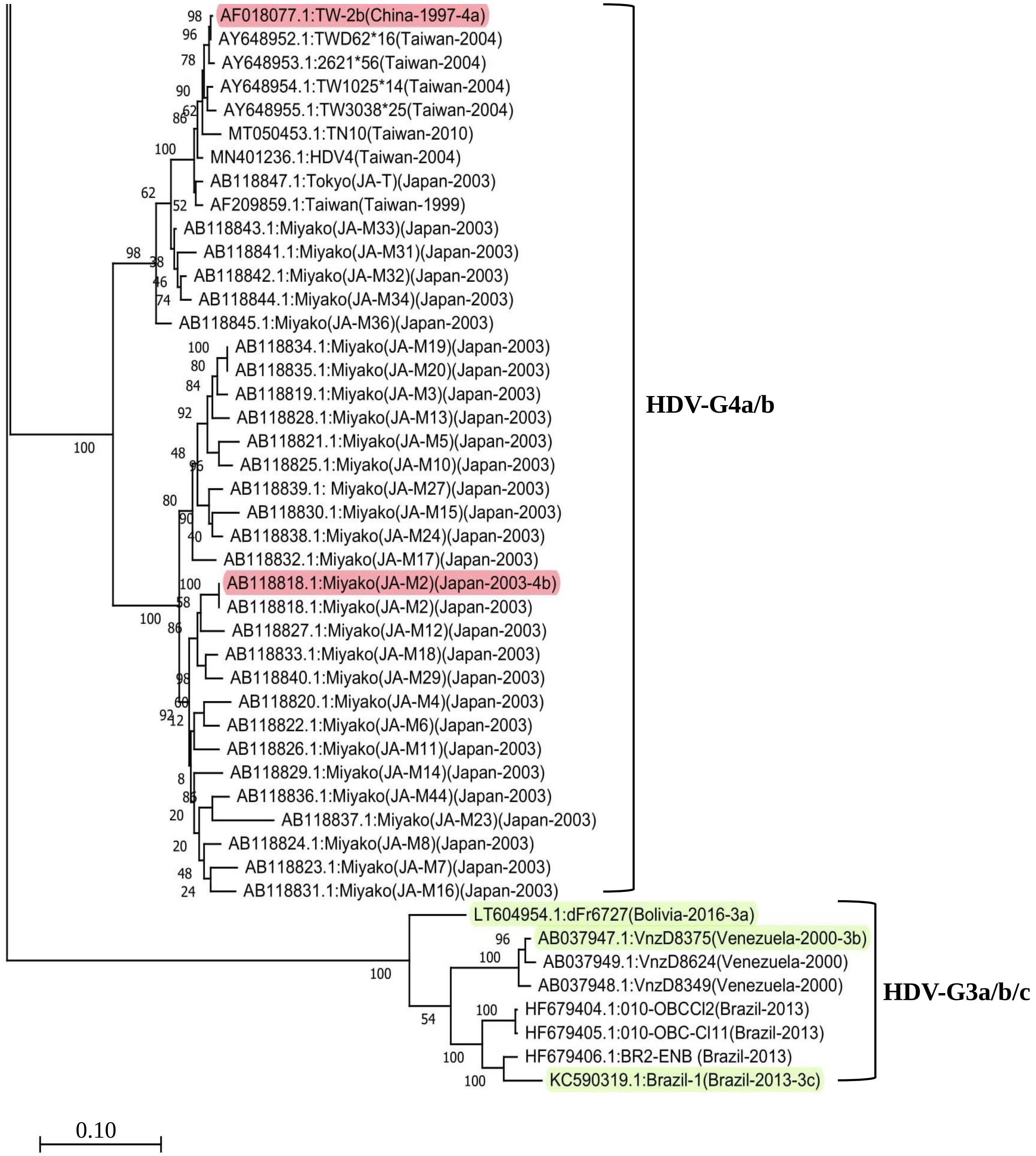

Supplementary Figure S6
